# Individual external doses below the lowest reference level of 1 mSv per year five years after the 2011 Fukushima nuclear accident among all children in Soma City, Fukushima: A retrospective observational study

**DOI:** 10.1371/journal.pone.0172305

**Published:** 2017-02-24

**Authors:** Masaharu Tsubokura, Michio Murakami, Shuhei Nomura, Tomohiro Morita, Yoshitaka Nishikawa, Claire Leppold, Shigeaki Kato, Masahiro Kami

**Affiliations:** 1 Department of Radiation Protection, Soma Central Hospital, Soma, Fukushima, Japan; 2 Department of Health Risk Communication, Fukushima Medical University School of Medicine, Fukushima, Fukushima, Japan; 3 Department of Global Health Policy, Graduate School of Medicine, University of Tokyo, Bunkyo-ku, Tokyo, Japan; 4 Department of Epidemiology and Biostatistics, School of Public Health, Imperial College London, Norfolk Place, London, United Kingdom; 5 Department of Internal Medicine, Soma Central Hospital, Soma, Fukushima, Japan; 6 Department of Health Informatics, School of Public Health, Kyoto University, Kyoto, Kyoto, Japan; 7 Global Public Health Unit, School of Social and Political Science, University of Edinburgh, Edinburgh, United Kingdom; 8 Research Institute of Innovative Medicine, Tokiwa Foundation, Iwaki, Fukushima, Japan; 9 Medical Governance Research Institute, Minato-ku, Tokyo, Japan; Northwestern University Feinberg School of Medicine, UNITED STATES

## Abstract

After the 2011 Fukushima Daiichi nuclear power plant accident, little information has been available on individual doses from external exposure among residents living in radioactively contaminated areas near the nuclear plant; in the present study we evaluated yearly changes in the doses from external exposure after the accident and the effects of decontamination on external exposure. This study considered all children less than 16 years of age in Soma City, Fukushima who participated in annual voluntary external exposure screening programs during the five years after the accident (n = 5,363). In total, 14,405 screening results were collected. The median participant age was eight years. The geometric mean levels of annual additional doses from external exposure attributable to the Fukushima accident, decreased each year: 0.60 mSv (range: not detectable (ND)–4.29 mSv), 0.37 mSv (range: ND–3.61 mSv), 0.22 mSv (range: ND–1.44 mSv), 0.20 mSv (range: ND–1.87 mSv), and 0.17 mSv (range: ND–0.85 mSv) in 2011, 2012, 2013, 2014, and 2015, respectively. The proportion of residents with annual additional doses from external exposure of more than 1 mSv dropped from 15.6% in 2011 to zero in 2015. Doses from external exposure decreased more rapidly than those estimated from only physical decay, even in areas without decontamination (which were halved in 395 days from November 15, 2011), presumably due to the weathering effects. While the ratios of geometric mean doses immediately after decontamination to before were slightly lower than those during the same time in areas without decontamination, annual additional doses reduced by decontamination were small (0.04–0.24 mSv in the year of immediately after decontamination was completed). The results of this study showed that the levels of external exposure among Soma residents less than 16 years of age decreased during the five years after the Fukushima Daiichi nuclear power plant accident. Decontamination had only limited and temporal effects on reducing individual external doses.

## Introduction

Radiation exposure can result in long-term health risks such as tumors, depending on personal doses.[1] Health threats have emerged in radioactively contaminated areas after nuclear accidents such as the 1986 Chernobyl accident.[2] Cumulative exposure is a serious public concern in Fukushima, particularly among children, who may face future health risks.[3]

In order to control doses of cumulative exposure following a major radiation-release accident, the reduction of chronic exposure, which accounts for the majority of the total radiation exposure, is of great importance.[2] Chronic exposure is classified as internal or external. While the total dose from both internal and external exposure is a crucial risk factor for radiation-related health outcomes,[1] there are several differences in characteristics between individual doses from internal and external exposure, including the distributions of doses, decreasing trends (i.e. half-life periods), high-risk behaviors for radiation exposure among residents in radioactively contaminated areas, and countermeasures such as food regulation and decontamination.[2] Internal contamination may continue for an extended period due to prolonged soil and subsequent food contamination.[4] The individual levels of internal contamination are strongly influenced by dietary habits, rather than by soil contamination levels in residential areas;[5, 6] thus, food regulation and identification of high-risk residents who regularly consume highly contaminated local food products are of particular importance.[7]

In contrast, individual doses from external exposure mainly depend on air dose rates in residential areas, and the locations where people daily spend long periods of time, while lifestyle habits themselves have limited impacts on the total doses from external exposure, especially among children.[8] Although decreasing trends in air dose rates are largely affected by the physical half-life of radioactive materials such as Cesium-134 (Cs-134) (2.0648 years) and Cs-137 (30.1671 years)—the major constituents of nuclear waste[9]—air dose rates in the environment decreased more rapidly than the physical decay of radioactive materials after the Chernobyl accident.[10] While several studies have evaluated the effect of remediation of radioactive contamination, such as decontamination in the case of the Chernobyl accident, decontamination efficiency varies depending on land use and air dose rate.[11–13]

After the Fukushima Daiichi nuclear power plant accident, which followed the Great East Japan Earthquake on March 11, 2011, internal exposure among residents in radioactively contaminated areas appears to have been marginal owing to quick and strict response by the Japanese central government and local authorities, including management of foodstuff contamination, which involved radiation inspection of foodstuffs circulating on the market and screening for internal contamination using whole-body counter (WBC) units.[14] While acute intake of radioactive materials immediately after the accident was the main contributor to internal exposure,[15] our series of publications found that, aside from those who deliberately and continuously consumed specific radioactively contaminated local foods, there were negligible chronic doses from internal exposure in Fukushima residents.[16–19]

However, in regards to the chronic external exposure among residents living in radioactively contaminated areas of Fukushima Prefecture, there is little information available on individual doses, especially yearly changes in doses from external exposure after the accident and the effects of decontamination on external exposure. Our previous study showed that doses from external exposure accounted for 90.3% of the chronic total doses in 2012 among residents following the Fukushima accident.[20] Thus, it is of considerable importance to evaluate the dose from external exposure. External doses can be estimated based on soil contamination or air dose levels.[21] Based on environmental monitoring data, external dose estimations after the Fukushima accident have been published by multiple authorities, including the World Health Organization (WHO)[22] and the United Nations Scientific Committee on the Effects of Atomic Radiation (UNSCEAR).[14] However, estimated doses may be higher than the actual individual dose measurements due to conservative hypotheses, and the fact that these estimations calculate representative effective doses of an average resident without considering inter-individual differences in radiation exposure and their resulting doses.[23] In fact, it has been found that external dose estimates based on air-dose measurement formulas recommended by the Japanese government have resulted in estimates three times higher than actual doses.[24, 25] While several studies have evaluated the efficiencies of decontamination after the Fukushima accident,[26–29] these assessments were based on the predicted external doses rather than individual measurements.

**Fig 1:**
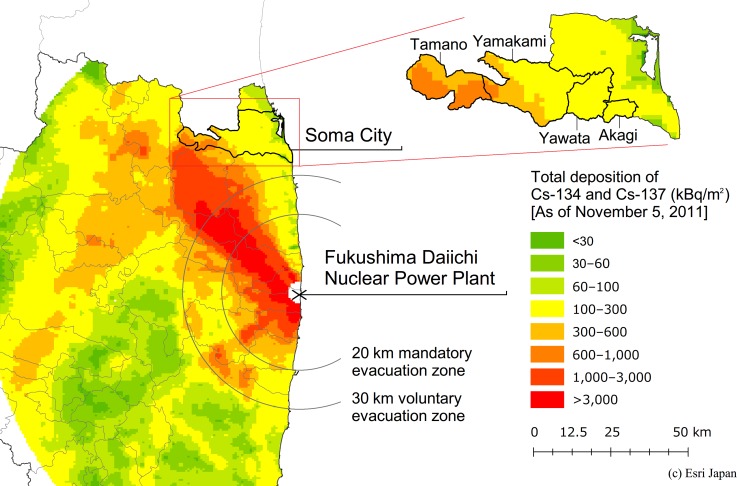
Soma City divided into four zones. Soma City is divided into eight administrative districts; as soil contamination levels differ by district, decontamination work has been performed in multiple areas. Zones 1, 2, 3, and 4 correspond to Yamakami, Yawata, Tamano districts and the Akagi area of Nittaki-district in Soma City, respectively.

Soma City is located 34–52 km from Fukushima Daiichi nuclear power plant and is adjacent to municipalities where evacuation was mandatory, including Minamisoma City (Fig 1). While the western parts of Soma City were highly radioactively contaminated with soil contamination of 13,000–900,000 Bq/m^2^ of Cs-134 + Cs-137 as of November 5, 2011, few people evacuated and most residents continued to live in the area after the Fukushima accident.[30] Thus, Soma City residents were ideal candidates for the assessment of external doses among those who continued to live in radioactively contaminated areas. Since 2011, Soma City has annually offered voluntary external radiation dose evaluations for all infants and students in elementary (6 to 12 years of age) and middle (13 to 15 years) schools using cumulative individual dosimeters, a useful method by which to assess individual external doses among all residents due to high participation proportions in the screenings.[31]

This study had two objectives. First, in order to evaluate the chronic levels of external exposure among residents in Soma City, we assessed the yearly changes in annual additional doses from external exposure individually for children less than 16 years of age using left-censored data. We also estimated ecological half-life and the length of periods in which annual additional doses measured in 2011 were halved. Second, to evaluate the efficacy of decontamination on individual doses from external exposure, we compared the decreasing trends in annual additional doses between areas where decontamination was and was not performed.

## Materials and methods

### Data and setting

In order to evaluate doses from chronic external exposure among residents near the crippled nuclear power plant, we analyzed data for all children less than 16 years of age in Soma City who participated in voluntary external exposure screening programs between October 2011 (the inception of the screening program) and November 2015. Screenings were performed annually for these five years after the Fukushima accident (see below for details about the screening program). All children who participated in any one of the annual screenings were enrolled in the present study. The collected data included participant age, sex, residential address at Ō-aza level—the most detailed level in the hierarchy of administrative division of city neighborhoods in Japan—and the screening results.

We divided Soma City into five zones (Zones 1, 2, 3, 4, and other areas (i.e. rest of the city)) depending on soil contamination levels and timing of decontamination. Zones 1, 2, 3, and 4 correspond to Yamakami, Yawata, Tamano districts and the Akagi area of Nittaki district in Soma City, respectively (Fig 1). These four Zones were the four highest radioactively contaminated areas in the city. Decontamination procedures were performed only in Zones 1, 2, 3, and 4. The decontamination was performed from December 2012 to November 2013 (Zone 1), August 2013 to March 2014 and July 2015 to November 2015 (Zone 2), August 2012 to December 2013 (Zone 3), and May 2014 to August 2014 (Zone 4), respectively. The decontamination procedures were administrated by Soma City and followed the standard regulations set by the Ministry of the Environment.[32]

### External exposure screening program

In response to the Fukushima nuclear accident, Soma City launched a voluntary external exposure screening program in October 2011 in cooperation with Chiyoda Technol Corp., Japan, a manufacturer of radiation protective products. This program is free of charge for infants, pregnant women who are registered in the Soma family registry, and all preschool toddlers, kindergarten children, and schoolchildren in Soma City. Notification of the program was sent to each school, and information was also disseminated using city magazines distributed to each household. The screening program was conducted in three-month cycles. First, the city office sent a radiation dosimeter (Glass Badge: GD-450, Chiyoda Technol Corp.) to the individuals or students’ guardians who consented to participate in the program. Screenings were performed annually between October 1, 2011, and November 30, 2015. The study periods were from October 1 to December 31, 2011 (1^st^ period), from July 1 to September 30, 2012 (2^nd^ period), from May 1 to July 31, 2013 (3^rd^ period), from September 1 to November 30, 2014 (4^th^ period), and from September 1 to November 30, 2015 (5^th^ period). During the screening, the participants were instructed to always carry their dosimeters, which measured their doses from external exposure, for three months. The instruction manual explains that the dosimeter needs to be hung from the user’s neck throughout the day, except when the user is at home, in which case the dosimeter can be placed near the user. The dosimeters were then returned to the city office.

Doses attributed to natural sources, including the universe itself (cosmic rays) and compounds within the earth, was subtracted from the measured values. These subtracted values were set as 0.21 mSv/year (0.024 μSv/h) from the universe and 0.33 mSv/year (0.038 μSv/h) from the earth, which themselves were the average values of the 20 Glass Badges with a 35-day measurement time at Oarai, Ibaraki Prefecture, Japan (located more than 100 km south of the Fukushima Daiichi nuclear power plant), before the accident. The range of measurement accuracy of the cumulative dose in 35 days was ± 4%.

The result of the Glass Badge measurement was expressed as a dose-equivalent at a tissue depth of 1 cm (Hp(10)). Hp(10) values obtained in the affected areas of Fukushima Prefecture are comparable to the effective dose of isotropic (ISO) or rotation (ROT) irradiation geometries.[33] While compliance with the proper use of Glass Badge was reported to be low among children, for example some participants were reported not to carry Glass Badge during the daytime; however, errors resulting from improper use of Glass Badge were very small and the method provides accurate evaluation of external exposure, as previously shown.[34] Thus, we regarded Glass Badge-measured doses to be equivalent to effective doses in the present study.

Therefore, the collected Glass Badge data were considered as an additional effective dose from external exposure after the Fukushima accident.

### Annual additional dose calculation

The annual additional doses from external exposure were estimated separately for each fiscal year between 2011 and 2015 (in Japanese, the fiscal year begins in April and ends in March) using the following methods. We first divided the three-month dose measurements for each year by the total measurement period (i.e. about 90 days), which were then multiplied by 365. Although external exposure generally includes a contribution from cloud-shine that is an important pathway just after an accident, it was not evaluated in the current study. We also did not consider several radioactive materials with short half-lives other than radioactive Cs, which were dispersed immediately after the accident. The estimated annual additional doses in 2011 were therefore likely underestimated. However, as the main objective of this study was to evaluate chronic doses from external exposure, we employed this calculation method.

### Calculation of mean value of annual additional doses

The detection limits of additional doses measured by a Glass Badge were 0.05 mSv/three months (< 0.2 mSv/y). This means that our original dataset was left-censored and that we did not have dose data for dose measurements that were below this limit (which were marked as ‘undetectable’). To evaluate the distributions of additional doses from external exposure in each year and their temporal reductions, left-censored datasets require statistical models, as developed in previous studies.[35, 36] According to these previous studies, we assumed that there were no zero values for the additional doses and that the dose data followed log-normal distributions. These assumptions allowed for estimation of the geometric means and standard deviations of the doses, including the left-censored data, for each year using a maximum-likelihood approach under the restriction that the estimated distribution of the dose data lower the detection limits were equal or higher than the observed negative ratios (ratios of left-censored data to total data).

### Calculation of the reduction ratios of annual additional doses

In order to assess the yearly changes in the annual additional doses from external exposure among children less than 16 years of age, we calculated the ratio and 95% confidence intervals (CI) of the estimated geometric mean values of annual additional doses from external exposure in each year to those in 2011 or to those in the previous year.

In order to evaluate ecological effects such as human activities and natural removal phenomena like weathering on reduction of doses, the reduction ratios were compared with those estimated from only physical decay.[37] The additional external dose following only physical decay was estimated as below (Eq 1).

$$D(t)=D(0)•\left\{0.73\times \exp\left(-\lambda_{Cs134}\times t\right)+0.27\times \exp\left(-\lambda_{Cs137}\times t\right)\right\}$$ (Eq.1)

where *D(t)* is the additional external dose at time *t*, *D(0)* is the additional external dose at time zero (As of August 23, 2011), and λ_Cs134_ and λ_Cs137_ are the physical decay constants (half-life times t_1/2-Cs134_ = 2.0648 y, t_1/2-Cs137_ = 30.1671 y; λ_Cs134_ = 0.0009191 d^-1^, λ_Cs137_ = 0.00006291 d^-1^).[9] “*t*” is the elapsed days from August 23, 2011.

The reference date of August 23, 2011 was selected, as was described in the previous study (contribution to additional effective dose, Cs-134: Cs-137 = 0.73:0.27 on August 23, 2011). [38]

### Estimation of the ecological half-life and the length of periods in which annual additional doses measured in 2011 were halved

To estimate the ecological half-life and the length of periods in which annual additional doses measured in 2011 were halved, we used the following method according to a previous study.[39] The prediction model for additional external dose in the environment can be expressed as follows:

$$\begin{array}{c} D(t)=D(0)•\{a_{fast}•\exp(\frac{-\ln\;2}{T_{efast}}•t)+(1-a_{fast})•\exp(\frac{-\ln\;2}{T_{eslow}}•t)\}\\ •\left\{0.73\times \exp\left(-\lambda_{Cs134}\times t\right)+0.27\times \exp\left(-\lambda_{Cs137}\times t\right)\right\} \end{array}$$ (Eq.2)

where *a*_*fast*_ is the fractional distribution of fast elimination component, *T*_*efast*_ is the ecological half-life for the fast elimination component, and *T*_*eslow*_ is the ecological half-life for the slow elimination component.

The slow component might not be explicit during the relatively short observation time of the present study, since *T*_*eslow*_ is estimated longer than several decades.[39] Thus, Eq 2 can be reduced as follows:

$$\begin{array}{c} D(t)=D(0)•\{a_{fast}•\exp(\frac{-\ln\;2}{T_{efast}}•t)+(1-a_{fast})\}\\ \;•\left\{0.73\times \exp\left(-\lambda_{Cs134}\times t\right)+0.27\times \exp\left(-\lambda_{Cs137}\times t\right)\right\} \end{array}$$ (Eq.3)

On the basis of Eq 3, we estimated the ecological half-life *T*_*efast*_ and *a*_*fast*_, by least-squares method, using the geometric mean values in areas without decontamination procedures (i.e., other areas). Based on these values we then estimated the length of periods, in which the additional external doses measured in November 15, 2011 (the median of the survey) were halved.

To support the validity of the above method, we also calculated the length of periods, in which the additional external doses measured in 2011 were halved using the following method.

In Kinase et al. (2014),[39] the authors also reported that *T*_*efa*st_ is given under assumption that doses can be expressed by mono-exponential functions as follows:

$$T_{efast}=\ln\;2•\frac{t_{2}-t_{1}}{\ln\left(\frac{Y(t_{1})}{Y(t_{2})}\right)}$$ (Eq.4)

where *t*_*1*_ is November 15, 2011 (median of the survey in 2011), *t*_*2*_ is June 15, 2013 (median of the survey in 2013), *Y(t*_*1*_*)* and *Y(t*_*2*_*)* are the geometric mean values of the additional external dose in areas without decontamination procedures at the year of 2011 and 2013, respectively, and decay-corrected to the value on November 15, 2011 following the Eq 1.

For this calculation, we have to choose 2 point as *t*_*1*_ and *t*_*2*_ from given 5 periods of external radiation screenings. Since the objective of this calculation was to determine the length of periods, in which the additional external doses measured in 2011 were halved, and the additional external doses in 2011 were decreased to be half between 2012 and 2013, we have used the GB data in 2011 and 2013

### Evaluation of the effects of decontamination procedures

To evaluate the effects of decontamination procedures, z-tests were performed to test for differences in the ratios of geometric mean values before and after the decontamination procedures (i.e. difference in log-transformed mean doses before and after decontamination) between individual zones and other areas (those without decontamination; control sites). Considering the decontamination periods described above, we used the ratios of doses in 2014 to 2012 for Zone 1 vs. other areas, 2015 to 2013 for Zone 2 vs. other areas, 2014 to 2011 for Zone 3 vs. other areas, and 2014 to 2013 for Zone 4 vs. other areas.

### Ethics

The Institutional Review Board of Minamisoma Municipal General Hospital approved the study (authorization number 28–02). For the use of external exposure data, the ethics committee agreed that written consent was not required for each participant in the present study.

## Results

The demographic characteristics of participants of the external exposure screenings in Soma City are shown in Table 1. In total, 14,405 measurements (no. of individual = 5,363) were performed in the five-year study period. The median age of the participants at the time of the screenings was eight years (range: 0–15). The sex of participants was unavailable in the database. The populations of residents aged less than 16 years in Soma City were 5,618, 5,350, 5,192, 5,113, and 5,002 from 2011 to 2015, respectively, and 37.0 to 71.5% of residents participated in the screenings each year. The numbers of participants decreased each year.

**Table 1:** Demographic characteristics of the participants of the external exposure screenings in Soma City.

	Year	2011	2012	2013	2014*	2015
Total participants (n = 14,405)		3,812	3,824	2,979	1,937	1,853
Age (years; median: range)		8 (0–15)	8 (0–15)	8 (1–15)	8 (0–15)	8 (0–15)
	Less than 6 (infants and pre-school)	1,320 (34.6)	1,221 (31.9)	988 (33.2)	302 (19.2)	584 (31.5)
	6–12 (Elementary school)	1,854 (48.6)	1,959 (51.2)	1,591 (53.4)	1,070 (68.0)	1,038 (56.0)
	13–15 (Junior high school)	638 (16.7)	644 (16.8)	400 (13.4)	201 (12.8)	231 (12.5)
Zones						
	1 (Yamakami-district)	157 (4.1)	144 (3.8)	123 (4.1)	75 (3.9)	56 (3.0)
	2 (Yawata-district)	231 (6.1)	205 (5.4)	177 (5.9)	125 (6.5)	119 (6.4)
	3 (Tamano-district)	23 (0.6)	18 (0.5)	12 (0.4)	10 (0.5)	11 (0.6)
	4 (Akagi-area in Nittaki-district)	26 (0.7)	27 (0.7)	22 (0.7)	7 (0.4)	8 (0.4)
	Other areas	3375 (88.5)	3430 (89.7)	2645 (88.8)	1720 (88.8)	1659 (89.5)

*Age data is unavailable for 364 participants in 2014.

### Annual additional doses from external exposure

**Fig 2:**
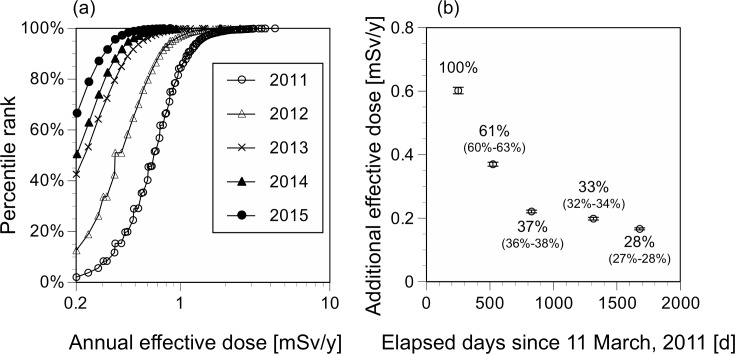
Trends in the annual additional doses from external exposure among children in Soma City. (a) Distribution; (b) geometric mean. The percentage values represent the ratio of the geometric mean dose (95% CI) in each year to that in 2011.

The trends in annual additional doses from external exposure among children in Soma City are shown in Fig 2(A). Estimation using left-censored data showed that the geometric mean levels of the annual additional doses from external exposure decreased each year: 0.60 mSv (range: not detectable (ND)–4.29 mSv), 0.37 mSv (range: ND–3.61 mSv), 0.22 mSv (range: ND–1.44 mSv), 0.20 mSv (range: ND–1.87 mSv), and 0.17 mSv (range: ND–0.85 mSv) in screening programs performed in 2011, 2012, 2013, 2014, and 2015, respectively (Table 2).

**Table 2:** Geometric mean levels of the annual additional doses from external exposure among children in Soma City.

	Year	2011	2012	2013	2014	2015
Whole, n		3,812	3,824	2,979	1,937	1,853
	Geometric mean dose (mSv/y)	0.60	0.37	0.22	0.20	0.17
	Geometric standard deviation	1.72	1.71	1.71	1.61	1.53
	ND, %	79 (2.1)	484 (12.7)	1,268 (42.6)	980 (50.6)	1,235 (66.6)
	More than 1 mSv/y, %	593 (15.6)	115 (3.0)	8 (0.3)	2 (0.1)	0 (0)
Zone 1, n		157	144	123	75	56
	Geometric mean dose (mSv/y)	0.82	0.58	0.29	0.27	0.21
	Geometric standard deviation	1.76	1.54	1.62	1.48	1.67
	ND, %	1 (0.6)	1 (0.7)	27 (22.0)	16 (21.3)	26 (46.4)
	More than 1 mSv/y, %	60 (38.2)	14 (9.7)	1 (0.8)	0 (0)	0 (0)
Zone 2, n		231	205	177	125	119
	Geometric mean dose (mSv/y)	0.93	0.60	0.34	0.29	0.22
	Geometric standard deviation	1.45	1.60	1.74	1.43	1.45
	ND, %	0 (0)	2 (1.0)	29 (16.4)	19 (15.2)	46 (38.7)
	More than 1 mSv/y, %	105 (45.5)	28 (13.7)	2 (1.1)	0 (0)	0 (0)
Zone 3, n		23	18	12	10	11
	Geometric mean dose (mSv/y)	2.53	1.59	0.74	0.60	0.40
	Geometric standard deviation	1.28	1.55	1.39	1.65	1.44
	ND, %	0 (0)	0 (0)	0 (0)	0 (0)	0 (0)
	More than 1 mSv/y, %	23 (100)	16 (88.9)	3 (25.0)	1 (10.0)	0 (0)
Zone 4, n		26	27	22	7	8
	Geometric mean dose (mSv/y)	0.83	0.59	0.42	0.35	0.29
	Geometric standard deviation	1.35	1.38	1.55	1.37	1.50
	ND, %	0 (0)	0 (0)	1 (4.5)	0 (0)	1 (12.5)
	More than 1 mSv/y, %	3 (11.5)	2 (7.4)	0 (0)	0 (0)	0 (0)
Other areas, n		3,375	3,430	2,645	1,720	1,659
	Geometric mean dose (mSv/y)	0.57	0.35	0.21	0.19	0.16
	Geometric standard deviation	1.69	1.67	1.64	1.59	1.48
	ND, %	78 (2.3)	481 (14.0)	1211 (45.8)	945 (54.9)	1162 (70.0)
	More than 1 mSv/y, %	402 (11.9)	55 (1.6)	2 (0.1)	1 (0.1)	0 (0)

Zones 1, 2, 3, and 4 correspond to Yamakami, Yawata, Tamano districts and the Akagi area of Nittaki-district in Soma City, respectively.

The proportion of residents with annual additional doses from external exposure above 1 mSv showed a clear declining trend each year, dropping from 15.6% in 2011 to 0% in 2015, and differed according to zone. In contrast, the proportion of residents with annual additional doses from external exposure below detection limits was 2.1% in 2011, and increased each year, to 66.6% in 2015. The proportion of residents below detection limits was 0% in Zone 3 throughout the study period; however differences existed between zones.

### Yearly changes in annual additional doses from external exposure

**Fig 3:**
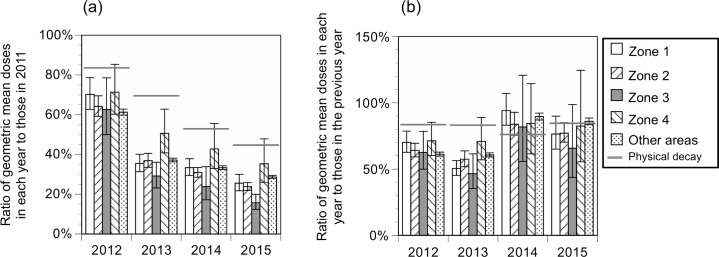
**Ratios of the geometric mean doses from external exposure in individual zones compared to 2011 (a) or the previous year (b); compared to doses estimated from the physical decay of radioactive Cs.** The error bars represent the 95% CI.

The reduction ratios of doses from external exposure in whole areas and individual zones in comparison to 2011 and in comparison to the doses estimated from the physical decay of radioactive Cs are shown in Figs 2(B) and 3(A). The ratios of the geometric mean doses from external exposure in each year to that in 2011 in whole areas decreased to 61% (95% CI: 60%–63%), 37% (36%–38%), 33% (32%–34%), and 28% (27%–28%), in 2012, 2013, 2014, and 2015, respectively.

The ratios of geometric mean doses from external exposure in each year to that of 2011 were significantly lower (i.e. reduction ratios were greater) in every zone (except Zone 4 in 2012, 2014, and 2015) than those estimated from only physical decay of radioactive Cs, which were 84%, 69%, 53%, and 45% in 2012, 2013, 2014, and 2015, respectively. However, the ratios of the geometric mean doses from external exposure in 2012 and 2013 to the previous year were lower (i.e., reduction ratios were greater), except for Zone 4, than those estimated from only physical decay (84% and 83%), while the ratios in 2014 and 2015 to the previous year were comparable to or higher than those estimated from only physical decay (76% and 85%) (Fig 3(B)). Details are described in the Supporting information (Tables A and B in S1 File).

### Ecological half-life and length of periods in which annual additional doses measured in 2011 were halved

From Eq 3, ecological half-life *T*_*efast*_ and *a*_*fast*_ was estimated as 123 days and 0.52, respectively, for areas without decontamination. Based on these values, doses from external exposure based on actual measurements in November 15, 2011 (the median of the survey) were halved in 395 days, thus shorter than those estimated from only physical decay (1,170 days). The length of periods, in which doses in November 15, 2011 were halved, was also calculated as 399 days from Eq 4, which well agreed with that estimated from Eq 3.

### Efficacy of decontamination

**Fig 4:**
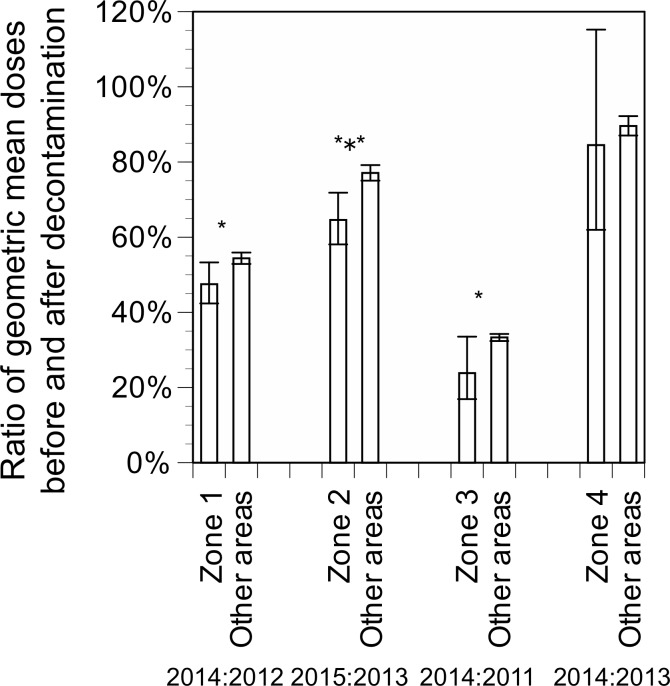
Ratios of geometric mean doses after decontamination compared to those before. * *P* < 0.05, *** *P* < 0.001. The top and bottom of the error bars represent the 95% confidence intervals.

The ratios of geometric mean doses after decontamination to before in Zones 1–3 were significantly lower (i.e., reduction ratios were higher) than those in other areas (Fig 4): *P* < 0.001 for Zone 2 vs. other areas (the ratios of geometric mean doses of 2015 to 2013: 65% vs. 77%); *P* < 0.05 for Zone 1 vs. other areas (2014 to 2012; 48% vs. 54%) and for Zone 3 vs. other areas (2014 to 2011; 24% vs. 33%), whereas no significant differences were found between Zones 4 and other areas (2014 to 2013; 85% vs. 90%) (*P* > 0.05). Decontamination contributed to reducing 13% of the additional effective doses in Zone 1, 16% in Zone 2, and 28% in Zone 3, respectively, although annual additional doses reduced by decontamination were small (0.04 mSv in the year of 2014 for Zone 1, 0.04 mSv in the year of 2015 for Zone 2, and 0.24 mSv in the year of 2014 for Zone 3, respectively).

## Discussion

The results of this study showed that the levels of external exposure among Soma residents less than 16 years of age decreased during the five years after the Fukushima Daiichi nuclear power plant accident, suggesting that the health effects from chronic external exposure were likely negligible. The levels of annual external exposure among all children less than 16 years of age in 2015, five years after the accident, were below 1 mSv, which is the lowest reference level under the existing exposure situation among the public in the band of 1 to 20 mSv per year following International Commission on Radiation Protection (ICRP) recommendation. [40] These findings indicate that doses from external exposure among all residents in Soma were also likely marginal, although a previous study showed that doses from external exposure among adults were generally higher than those among children since adults tended to spend more time in radioactively contaminated areas.[41] The effective dose from external exposure in the first year of the accident among 10-year-old Soma City children estimated by UNSCEAR was higher than that estimated in the present study (0.97 mSv vs. 0.60 mSv).[14] While we did not evaluate doses due to external exposure immediately after the accident, which included contributions from cloud-shine and from short half-life radioactive materials other than radioactive Cs, in the present study, the UNSCEAR report showed that the doses from cloud-shine were negligible (~0 mSv) in this area. The difference may be due to external exposure, including short half-life radioactive materials, during the very early phase of the accident or differences in assumptions in the UNSCEAR report (e.g. shielding properties and the amounts of time spent in different types of location) and actual conditions.

Doses from external exposure decreased more rapidly compared to those estimated based on the physical decay of radioactive materials even in areas without decontamination. Doses from external exposure in November 15, 2011 (the median of the survey) were halved in 395 days, which is shorter than that estimated from only physical decay (1,170 days). Previous studies proposed that air dose rates in the environment decreased more rapidly than the physical decay of radioactive materials,[10] which is consistent with the results of the present study. Interestingly, the reduction ratios in 2012 and 2013 compared to the previous year were greater than those estimated from only physical decay in almost every place, including areas without decontamination (‘other areas’); however, the reduction ratios in 2014 and 2015 compared to the previous year were comparable to or even smaller than those estimated from only physical decay. These findings are presumably attributable to the fact that reductions of dose from ecological effects (e.g, weathering) occur mainly during the early stages of a nuclear accident (i.e. within a few years of the accident) and not after a long period of time. While physical decay of radioactive materials and weathering likely affected the decreased doses from external exposure in the present study, personal countermeasures such as avoidance of potentially radioactively contaminated areas and reducing time outdoors may have also contributed to the decreased doses. While the efficacies of individual countermeasures differ depending on various factors including soil contamination levels of residential areas and individual demographic backgrounds, future studies are warranted to evaluate which countermeasures are most effective to reduce chronic external doses among residents.

The results of this study also suggest the limited effects of decontamination on reducing individual external doses. Although the reduction ratios of doses after decontamination to their levels before in Zones 1–3 were significantly higher than those in other areas without decontamination in the present study, annual additional doses reduced by decontamination were small (0.04 mSv in the year of 2014 for Zone 1, 0.04 mSv in the year of 2015 for Zone 2, and 0.24 mSv in the year of 2014 for Zone 3, respectively). These findings suggest that decontamination led to a temporal reduction of external doses right after the decontamination, yet seemed have little impact on cumulative effective doses and on radiation-related health outcomes.

Of note, the reduction ratios due to decontamination were the highest in Zone 3, where air dose rates were the highest among the zones and decontamination began at the earliest stage. There are several possible explanations for these differences in the efficacy of decontamination among zones in the present study. First, differences in soil type and in principal uses of the land, such as urban areas and farmland, between zones might have contributed to the observed differences. [42] Second, the efficacy of decontamination depends on the levels of soil contamination and increases with increased dose rates, as shown in a previous study.[26] This result highlights that decontamination procedures, if necessary, should be applied in the early stage of a nuclear accident when air dose rates are at higher levels. Third, personal countermeasures among residents in Zones 1–3 and other areas may have differed during the study period.

## Limitations

The present study has limitations. First, soil contamination levels in Soma City after the Fukushima accident were 13,000–900,000 Bq/m^2^ (as of November 5, 2011). The absolute values of the reduction ratios of individual exposure may depend on the soil contamination level in living areas, and our findings with regard to the efficacy of decontamination on individual doses are not generalizable to other areas with higher contamination levels in Fukushima Prefecture. Second, because the study participants were children in Soma City whose daily lifestyles and living environments might differ from those of other population groups, we cannot generalize the results of this study to the whole population in Fukushima. Third, this study was voluntary; those with concerns about radiation exposure might have been more likely to participate and thus be included in the study. Considering that individuals concerned about radiation might be more likely to undertake countermeasures to reduce exposure levels in their daily lives, which could not be monitored nor adjusted for in the analyses, our study may have been affected by selection bias.

## Conclusions

The results of this study showed that the levels of external exposure among Soma residents less than 16 years of age decreased during the five years after the Fukushima Daiichi nuclear power plant accident and that the health effects from chronic external exposure were likely to be negligible. Doses from external exposure in November 15, 2011 (the median of the survey) were halved in 395 days for areas without decontamination, which was much shorter than those estimated from only physical decay. Decontamination may have limited effects on reducing individual external doses.

## Supporting information

S1 File Table A. Ratios of geometric mean doses from external exposure in individual zones in comparison to 2011. Table B. Ratios of geometric mean doses from external exposure in individual zones in comparison to the previous year.
(DOCX)
