# Supplementary material for: Individual external doses below the lowest reference level of 1 mSv per year five years after the 2011 Fukushima nuclear accident among all children in Soma City, Fukushima: A retrospective observational study
Source: PLoS One. 2017 Feb 24;12(2):e0172305. doi: 10.1371/journal.pone.0172305 (PMC5325236; doi:10.1371/journal.pone.0172305)
Supplement: S1 File — Table A. Ratios of geometric mean doses from external exposure in individual zones in comparison to 2011. Table B. Ratios of geometric mean doses from external exposure in individual zones in comparison to the previous year. (DOCX) [file pone.0172305.s001.docx]

**Table A. Ratios of geometric mean doses from external exposure in individual zones in comparison to 2011.**

|  | 2012 | 2013 | 2014 | 2015 |
| --- | --- | --- | --- | --- |
| Zone 1 | 70% (63%-79%) | 35% (31%-40%) | 33% (29%-38%) | 26% (22%-30%) |
| Zone 2 | 64% (59%-70%) | 37% (34%-41%) | 31% (29%-33%) | 24% (22%-26%) |
| Zone 3 | 63% (50%-79%) | 29% (23%-36%) | 24% (17%-34%) | 16% (12%-20%) |
| Zone 4 | 71% (60%-85%) | 51% (41%-63%) | 43% (33%-56%) | 35% (26%-48%) |
| Other areas | 61% (60%-63%) | 37% (36%-38%) | 33% (32%-34%) | 29% (28%-29%) |
| Physical decay only | 84% | 69% | 53% | 45% |

Values in parenthesis represent 95% CI.

**Table B. Ratios of geometric mean doses from external exposure in individual zones in comparison to the previous year.**

|  | 2012 | 2013 | 2014 | 2015 |
| --- | --- | --- | --- | --- |
| Zone 1 | 70% (63%–79%) | 51% (45%–56%) | 94% (83%–107%) | 77% (65%–90%) |
| Zone 2 | 64% (59%–70%) | 57% (52%–64%) | 84% (76%–93%) | 77% (70%–85%) |
| Zone 3 | 63% (50%–79%) | 47% (35%–62%) | 82% (56%–121%) | 66% (44%–99%) |
| Zone 4 | 71% (60%–85%) | 71% (57%–89%) | 85% (62%–115%) | 82% (55%–124%) |
| Other areas | 61% (60%–63%) | 61% (59%–62%) | 90% (87%–92%) | 86% (84%–89%) |
| Physical decay only | 84% | 83% | 76% | 85% |

Values in parenthesis represent 95% CI.
